# Investigating Muscle Activation Patterns and Muscle Synergies Pre- and Post-Balance Training in Older Adults

**DOI:** 10.3390/app15116151

**Published:** 2025-05-29

**Authors:** J’niya Butler, Ambika Bhatta, Nicole Arnold, Younes El Hakour, Lara A. Thompson

**Affiliations:** Center for Biomechanical & Rehabilitation Engineering, School of Engineering and Applied Sciences, University of the District of Columbia, 4200 Connecticut Ave. NW, Washington, DC 20008, USA

**Keywords:** gait analysis, balance, electromyography (EMG), elderly, stroke, muscle synergy

## Abstract

In the aging population, imbalance leading to falls is of critical concern; thus, it is imperative to determine and quantify neuromuscular changes because of rehabilitative balance training. (1) Background: Previous studies that have examined changes in balance due to rehabilitative training placed a focus on clinical measures (i.e., behavioral, kinetic, and kinematic outputs); however, irregularities due to abnormalities of underlying neural origin were unrevealed by the aforementioned measures. (2) Methods: Examining muscle activity was used to determine strategies pre- and post-six weeks of balance training in twenty-three healthy older adults (69.5 ± 5.7 years old) and five survivors of stroke (66.4 ± 9.48 years old). Surface electromyographic (sEMG) signals were recorded from eight of the lower limb muscles while participants performed forward walking (FW), forward tandem walking (FT-W), backward walking (BW), and backward tandem walking (BT-W) tasks. The sEMG data were then conditioned and muscle synergies were extracted using non-negative matrix factorization (NNMF). (3) Results: It was observed that muscle synergies and activation patterns changed for pre- versus post-balance training in older adults (i.e., healthy individuals and those who had suffered from stroke). (4) Conclusions: From our findings, it was indicative that muscle activation and muscle synergies could be used to quantify and inform rehabilitative balance training in older adults.

## 1. Introduction

Aging leads to neuromuscular control difficulties while performing balance and walking needed for daily living [1]. With decreases in balance comes an increase in risk for falling leading to injury and even death; in the United States, one in four adults over 65 years old falls each year [2]. Aging is often accompanied by degeneration of nerve and muscle tissues [3] and of the sensory systems used for balance (i.e., visual, somatosensory, and vestibular), e.g., Ref. [4]. Moreover, aging is associated with major changes in the neuromotor system. In particular, these include reduction in muscle strength, power, and joint mobility along with an impaired sensorimotor integration [2]. Within the aging population, strokes are a prominent issue that can lead to disability and even death; over 60% of all strokes occur above the age of 65 years old [5]. Thus, effective rehabilitation is important towards facilitating balance maintenance in both older individuals that consider themselves healthy and survivors of stroke. Unfortunately, only 60 to 70 percent of survivors of stroke are able to walk again independently after rehabilitation [6].

In terms of quantifying balance and walking, common clinical measures have focused on qualitative tests, e.g., the commonly known Sit-to-Stand test and Timed Up and Go test (TUG), which can only offer crude, global, and/or descriptive information such as the time it takes to complete sitting and walking tasks, respectively. While kinematic measures (e.g., motion capture) can provide more detailed information in terms of movements, it is difficult to distinguish differences due to neural or musculoskeletal deficits. Further, forceplate methods (e.g., to measure center of pressure (COP)) may have limited applicability tied to movement due to the activation of different neural pathways because the same force and resulting movement can be achieved by many different muscle coordination patterns [7].

Muscle activation and muscle synergy analysis facilitate the characterization of neuromuscular control strategies for movement. Surface electromyography (sEMG) is a commonly used, non-invasive measure to determine muscle activation. Through postprocessing analysis of the raw sEMG signals, muscle synergies then can be determined tied to the activity of the muscles; thus, muscle synergy analysis can facilitate the characterization of neuromuscular control strategies and functional deficits [1,8,9]. Via spatial and temporal activation examined from the recorded muscle activity, muscle synergies offer insight in terms of muscle coordination and activation patterns. Patients with impaired motor functions tend to show an adaptation of muscle synergies to typical characteristics of movement (such as type of movement, speed, compensation of asymmetry due to neurological damage) [10]. After a stroke, fewer synergies are used during walking and upper-extremity tasks compared with unimpaired adults, possibly reflecting a simplified control strategy [11,12]; however, it will be interesting to determine if the same holds true for lower-extremity tasks such as walking. To the best of our knowledge, muscle synergy changes in older adults following several weeks of balance training have not been published, neither for healthy older adults nor survivors of stroke within the aged population.

Previous research regarding muscle synergies in humans has evaluated sEMG responses for walking and during platform perturbations, e.g., Refs. [1,12–17]. The effects of directionality tied to muscle synergy have been examined (e.g., during perturbations for angles between 0 to 270 degrees), e.g., Refs. [15,17]. While directional perturbation approaches provide insight into how the central nervous system affects muscle synergies via muscle tuning curves, these stimuli are not a direct comparison and atypical for movement one experiences in daily living. Task-specific muscle synergies have been examined (e.g., by performing straight forward walking, as well as right and left turning) [9]. However, the above studies have not considered quantifying the effects of balance training on muscle synergies (neither for healthy nor impaired older adults). Further, investigating the effects of more challenging balancing activities (e.g., tandem or backward walking) to evaluate the possibility of muscle synergy ‘improvement’ after training has not been determined. Although older adults could significantly decrease fall risk after balance training, neuromuscular metrics to determine which participants benefit from balance interventions are limited.

Here, by investigating neuromotor control during walking, we sought to understand real-life function and mobilization in the older population, as well as the impacts of balance training on both healthy older adults and older survivors of stroke. By examining whether muscle activation patterns and/or muscle synergies change following balance training (rehabilitation), we hypothesized that we could quantify and validate improvement of lower-limb motor function at the neural level in older adults. More specifically, we would see changes of decreases or increases in the number of synergies that reflect simplification or more complex control, respectively. Thus, we provide a proof of concept that muscle synergy analysis could be useful in comparing different groups of older adults’ balance training and determining if participants have gained motor functions that could generalize to activities of daily living, such as walking.

## 2. Materials and Methods

All study activities were conducted within the Center for Biomechanical and Rehabilitation Engineering (CBRE) laboratory at the University of the District of Columbia (UDC), the protocol was approved by the UDC Institutional Review Board (protocol no. 979744-1), and all participants gave their informed consent prior to participating in the study.

### 2.1. Participants

Participants were recruited for the study through flyers posted around the university and word of mouth. The population we were targeting were older individuals that considered themselves healthy and those who have suffered from stroke and were at least 6 months post-stroke. Our results are presented for 5 stroke (66.4 ± 9.48 years old) and 23 healthy participants (69.5 ± 5.7 years old). Participant demographics are shown in Table 1 (healthy) and Table 2 (survivors of stroke).

### 2.2. Training

Participants underwent a 6-week exercise routine which consisted of two 30 min sessions/week. During the sessions, the participants worked with the principal investigator and trainers (research assistants) who also served as spotters to support participants throughout the study as well as the NaviGAITor (multidirectional partial bodyweight support system) to prevent falls. This system did not provide bodyweight support but instead protected the participant in the event of a misstep or fall.

For the balance training sessions, each participant was worked with individually within the CBRE laboratory. Exercises were selected that required both healthy and stroke participants to make use of diverse sensory information to maintain their balance. During weeks one and two, participants performed walking exercises under both eyes-open and eyes-closed conditions, including forward and backward walking with both wide and tandem stepping. Weeks three and four focused on exercises conducted on foam surfaces of varying densities, incorporating standing balance tasks, isolated leg exercises, squats, and walking on hard, dense foam, or thick compliant foam surfaces. In weeks five and six, the training regimen combined walking over obstacles with additional foam-based exercises, further increasing sensory challenges and task complexity. The training consisted of various exercises that challenged visual, somatosensory, and vestibular systems to affect balance; vision was modified to make surroundings more or less challenging (eyes closed/eyes open, respectively), as well as supported surface somatosensory cues (hard or foam surfaces), while vestibular systems were highlighted by limiting combined visual and/or somatosensory cues (e.g., eyes closed while walking or standing on foam) [18]. Additionally, the base of support (BOS) was modified between large and small (e.g., double-leg, tandem, and single-leg stances) to increase task difficulty. For example, a larger BOS (double-leg stance) allows for increased stability whereas a smaller BOS (tandem and single-leg stances) could lead to decreased stability.

### 2.3. Assessment

**Figure 1.**
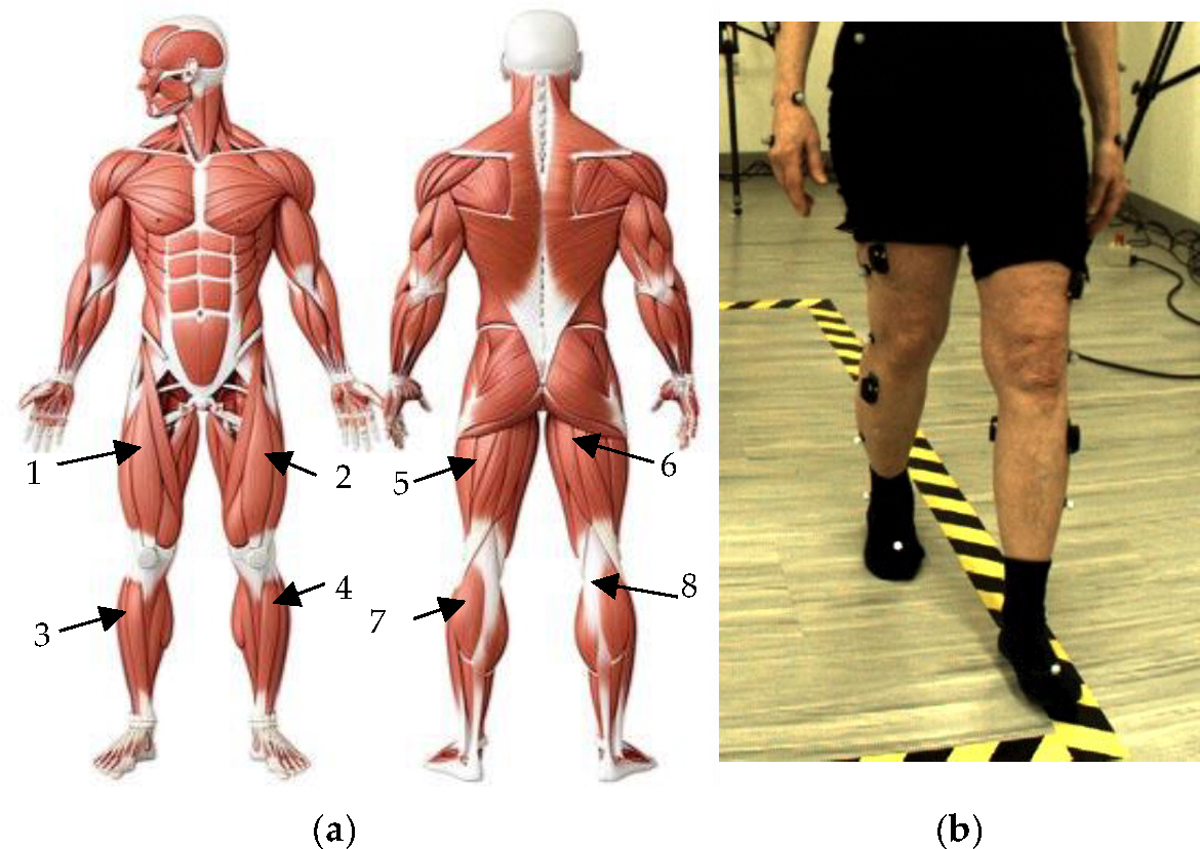
Locations of sEMG sensors: (**a**) Anatomical muscular system describing the location of each sEMG sensor placed on all participants’ (1) right and (2) left rectus femoris (RF), (3) right and (4) left tibialis anterior (TA), (5) left and (6) right bicep femoris (BF), and (7) left and (8) right medial gastrocnemius (MG) [19]; (**b**) example of participant performing forward walking task while donning EMG sensors.

Prior to data collection, a light sponge was used to remove any surface layers of dead skin, then each participant’s skin was cleansed with alcohol wipes prior to attachment of the EMG sensors, and lastly each sensor was affixed with strong double-sided tape (provided by Delsys). Eight wireless Delsys surface electromyography sensors (sEMG) (Trigno, Delsys, Boston, MA, USA) were placed on each muscle: the right and left rectus femoris (RF), right and left tibialis anterior (TA), right and left bicep femoris (BF), and right and left medial gastrocnemius (MG) (Figure 1). The sEMG data were collected using the Delsys Trigno System at a sampling frequency of 2000 Hz; the sEMG data was recorded, simultaneously with motion capture data, via Vicon Nexus 2.0 software.

Each participant completed a pre-assessment (at 0 weeks) and post-assessment (at 6 weeks) once the training was completed. Each participant performed forward and backward walking trials (FW and BW, respectively), in addition to forward and backward tandem walking trials (FT-W and BT-W, respectively), for a distance of 3 m, two trials per condition, per participant. Tandem walking is described as walking with one foot directly in front of the other, or heel to toe. For all walking trials, there was overground, unassisted walking and the participants were instructed to walk at their own, self-selected moderate pace.

### 2.4. Data Analysis

#### 2.4.1. Preprocessing and Conditioning Raw sEMG Data

After data acquisition was complete, raw data from the 8 EMG sensors were exported to .csv files for each participant for each walking condition. Each .csv file then displayed the EMG output in terms of microvolts as a function of time. The sEMG data were imported to MATLAB R2024b (MathWorks, Natick, MA, USA) for further processing. The sEMG data underwent analysis and processing using custom MATLAB (version 2024b, Matick, MA, USA) files inspired by analyses performed in previous studies, e.g., Refs. [1,20,21]. The preprocessing phase involved filtering muscle synergies using the raw EMG data. For the notch filter, built in function, “NotchPeakIIR()” with a center frequency of 40 Hz/sampling frequency, was implemented. The filtered output is again passed through a highpass filter using the “highpass()” function followed by a Butterworth lowpass filter, using the “filter()”function, where the 3rd-order Butterworth filter parameters are estimated from the function “butter()” using twice the center frequency as that of the notch filter. After highpass filtering the output undergoes demeaning and rectification. At the final step, the normalized response is interpolated, using the function “interp1()”, to maintain the uniform sampling of signal where 20 subframes for each time frame were recorded in the raw signal.

#### 2.4.2. Determining Muscle Synergies: Non-Negative Matrix Factorization (NNMF)

Muscle synergies were identified using non-negative matrix factorization (NNMF). NNMF motor module analysis of muscle coordination [17,21] was implemented here but adapted to suit the specific context of our study. NNMF is a technique that assumes the set of measured data is composed of linear combinations of a smaller number of underlying elements. The NNMF process represents the original sEMG data as a linear combination of vectors called muscle synergies (W) and activation coefficients (H) as shown in Equation (1).


(1) $$M=WH=W(m\times n)H(n\times t)=\sum_{n=0}^{7}\;W_{mn}H_{nt}$$

**Figure 2.**
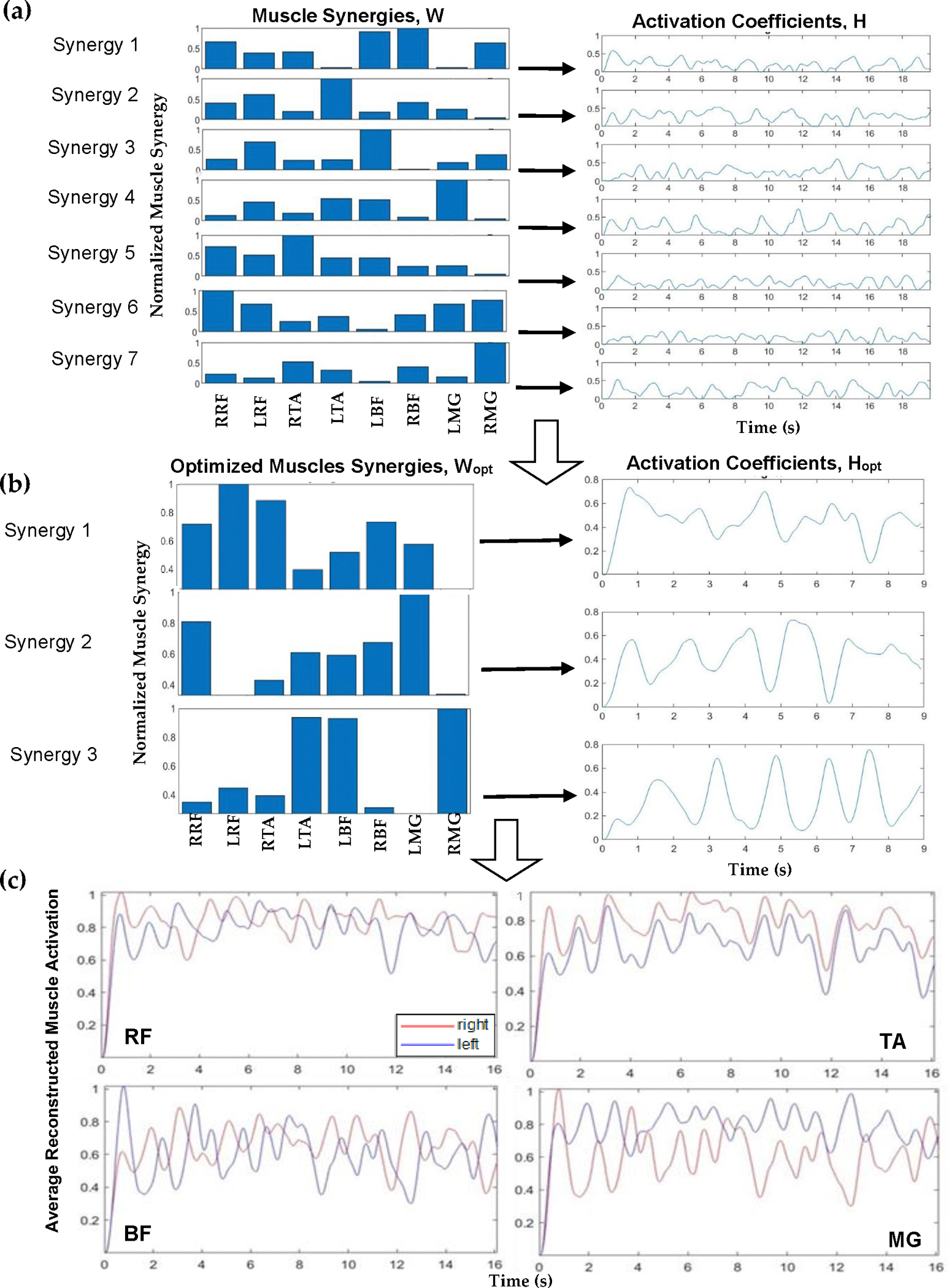
Schematic showing data processing workflow involving the following: (**a**) muscle synergies (W (left)) and activation coefficients (H (right)) for eight muscle groups; (**b**) optimized muscle synergies (W_opt_ (left)) and corresponding activation coefficients (H_opt_ (right)) for each optimized muscle synergy; and (**c**) normalized average reconstructed muscle activation for rectus femoris (RF), tibialis anterior (TA), bicep femoris (BF), and medial gastrocnemius (MG), right side (red) and left side (blue).

The matrix M defines spatial patterns of muscle activation across multiple muscles, or muscle synergies. This matrix is represented as the W matrix, where m is the number of muscle EMGs recorded (in this case 8) represented as rows and n is the number of synergies represented as columns. The values of H are between 0 and 1 as they are coefficients for normalized data. The matrix H describes the activation of the muscle contributions (or coefficients) over time, where n is the number of synergies represented as rows and t is the duration of each trial in samples represented as columns. The resulting (reconstructed) muscle activation, M, is then predicted. A schematic is shown in Figure 2.

**Figure 3.**
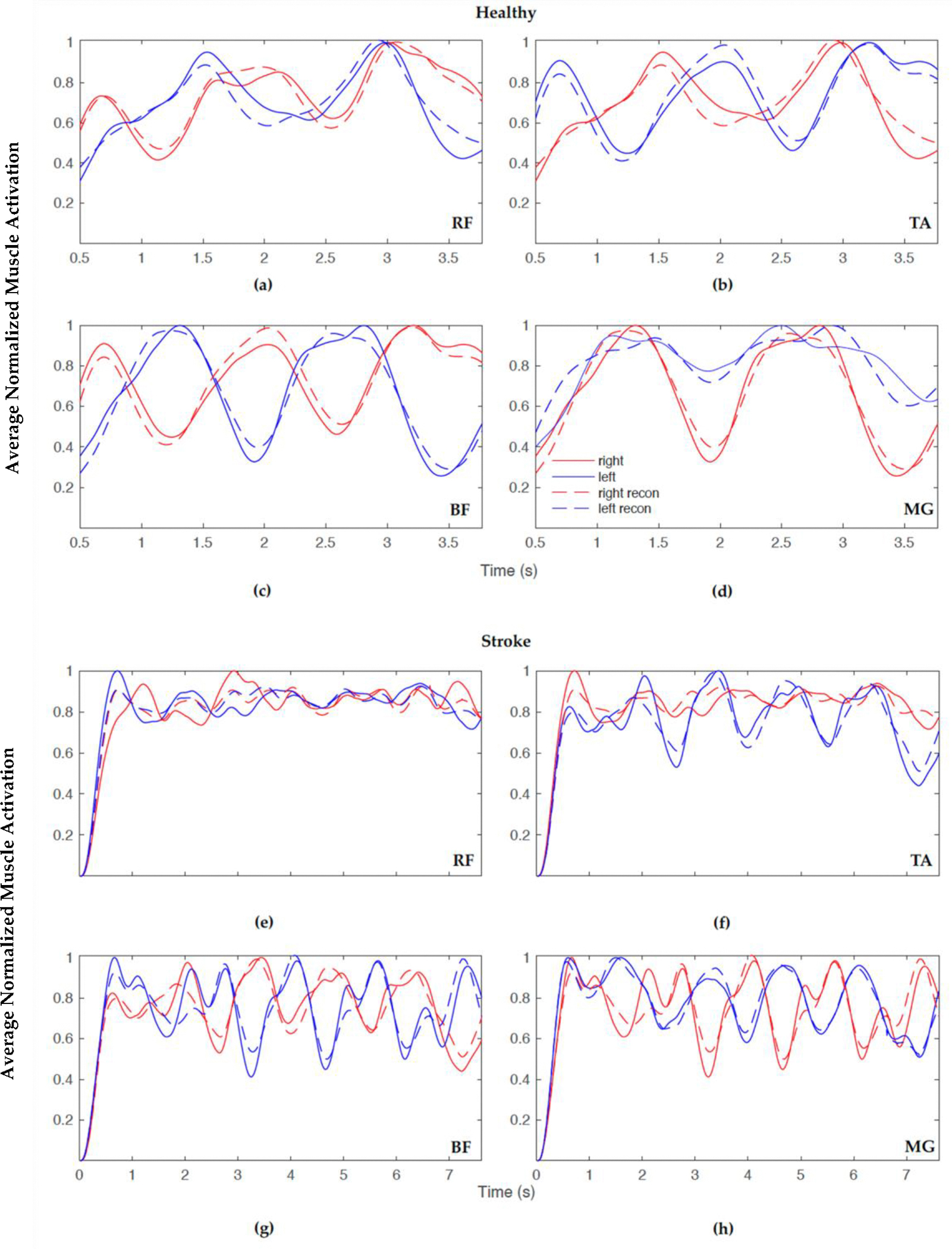
Example overlay plot of normalized averaged reconstructed muscle activation for healthy (top) and stroke (bottom) FW showing actual (solid) and predicted (dashed) with 98% VAF for the following: (**a**,**e**) rectus femoris (RF), (**b**,**f**) tibialis anterior (TA), (**c**,**g**) bicep femoris (BF), and (**d**,**h**) medial gastrocnemius (MG), right side (red) and left side (blue).

Implementing NNMF began with using a search algorithm with MATLAB, starting with a set of muscle synergies (where n is the number of muscle groups, and the max number of muscle synergies is n−1). The normalized synergy vectors and the corresponding weight coefficient were estimated using the MATLAB “nnmf()” function. For example, a schematic for one participant, FW pre-training, is shown in Figure 2. Figure 2a (left) displays muscle synergies represented for linear combination with the activation coefficients (Figure 2a, right) corresponding to each muscle, prior to this iterative approach. An iterative approach, varying between 1 and 7 synergies, was used to determine the appropriate number of muscle synergies to reconstruct the EMG data. The >98% variability accounted for the (VAF) threshold with >90% confidence interval (CI) for each muscle data vector guided the computation to yield the number of optimum muscle synergies, W_opt_, to represent and reconstruct the muscle activation data (Figure 2c). Muscle synergy values at this threshold were deemed valid to reconstruct the data. For the 98% benchmark, for example, three components were considered applicable to reconstruct the data as seen (Figure 2b). In this step, NNMF was further applied to the EMG data and reconstructed with three (optimal) muscle synergies (Figure 2b, left) and activation coefficients (Figure 2b, right). The reconstructed muscle responses are the resulting net activation of each muscle by all the muscle synergies recruited during each movement task (Figure 2c). As an example, a comparison of actual muscle activation and reconstructed muscle activation using 98% VAF is shown in Figure 3. A MATLAB code was generated to compute and plot the muscle synergy data over the average time it took to complete two gait cycles for each group (healthy or stroke) for each walking condition (FW, FT-W, BW, BT-W). Reconstructed synergies were computed for healthy and stroke participants for two gait cycles. When pooling results, if the participants completed two gait cycles in less or greater than the average time, the *interpol1* function in MATLAB was used to resample the synergies.

### 2.5. Statistical Analysis

Statistical analysis was performed using MATLAB. To obtain the differences between individual muscle synergies, the outliers and distribution of each synergy across participants of each walking condition were determined. Box plots were generated to examine medians, interquartile ranges, and outliers for each muscle group. Further, significance was determined by implementing the Kruskal–Wallis significance test; this test does not assume data are normally distributed. Optimizing the number of muscle synergies for fixed VAF also helped to identify the synergy with the highest variance. The first synergy for all sensors was utilized for the Kruskal–Wallis significance test function in MATLAB to obtain muscle activation *p*-values between pre- and post-training. Additionally, the Kruskal–Wallis test was implemented using RStudio software (RStudio Version 2023.12.1, Boston, MA, USA) to test for significant differences between the optimal number of muscle synergies for all groups and walking conditions, and pre vs. post *p*-values < 0.05 indicated significant differences. Cohen’s *d*, or the standardized mean difference, was implemented for the stroke participant group due to the small sample size. Cohen’s *d* specifically measures the effect size of the difference between two means.

## 3. Results

Our results showed changes in pre- compared to post-training as well as differences for healthy compared to stroke adults in terms of muscle activation and muscle synergy patterns, as described below in Sections 3.1 and 3.2, respectively.

### 3.1. Muscle Activation

Muscle activation pre- and post-training was examined for healthy (Table 3) and stroke (Table 4) participants. For healthy participants (FW and BW), the right RF (quad muscle) showed significant increases post-training. Further, for the healthy group, the right BF and the right MG exhibited significant increases in activation for FW. For the stroke group, the right TA showed significant increases post-training for BT-W. Also, for the stroke group, the right BF (FT-W) and left MG (BW) also showed significant increases.

Due to the smaller number of stroke participants, we also calculated Cohen’s *d*, as shown in Table 5. This measurement was calculated via the mean differences between the pre- and post-training means divided by the pooled standard deviation. A lower Cohen’s *d* indicates the necessity of larger sample sizes.

Table 5 shows the effect size calculations using the Cohen’s *d* equation. Cohen classified effect sizes as small (d = 0.2), medium (d = 0.5), and large (d ≥ 0.8); the larger the effect size, the more powerful it is. Using this calculation, the effect size quantifies the difference between two means. A smaller effect size indicates that the means between the pre- and post-training values (e.g., 0.3) only differ by 0.3 standard deviations, so the differences are minimal. A medium effect size (e.g., 0.6) indicates that the two means differ by 0.6 standard deviations, which can be considered non-trivial, but significance may be questioned. A larger effect size (e.g., >0.8) signifies a larger difference between the means and is confirmed by the results of the *p*-value. A smaller Cohen’s *d* value reported in this study indicated the potential need for a larger sample size required to make statistically significant differences; however, medium and large Cohen’s *d* values were observed in both forward and backward walking conditions.

### 3.2. Muscle Synergies

The 98% VAF led to the actual and reconstructed muscle activation patterns closely matching one another (as in Figure 3); however, Tables 6 and 7 display the average number of synergies for 80%, 90%, and 98% VAF with a constant confidence interval of 90% for healthy and stroke participants, respectively. In order to include all information from the EMG sensors and not to compromise accuracy, the choice was made to further examine synergies with a higher VAF (98%).

Table 8 displays the average number of muscle synergies and standard deviations for 98% VAF used for each group for each walking condition. From the results, it is observed that there were increases, on average, in the healthy group from easier (FW) to most difficult (BT-W) conditions. It is important to note that for BT-W (the hardest walking condition), for stroke, only the participants who felt the most comfortable and confident with their walking ability attempted; others opted out or could not complete it. Conversely, within each walking condition, there were slight decreases in numbers of synergies used post-compared to pre-training. For healthy adults, there were decreases in synergies, on average, post- compared to pre-training. Further, for BW and FT-W in the stroke group, there were decreases in the number of synergies, whereas for FW and BT-W there were increases in the number of synergies post- compared to pre-training.

Table 8 above shows the average number of muscle synergies for each walking condition within each group and their standard deviations in parentheses. For data shown in the above table, we compared the number of muscle synergies 1) within each group (healthy pre- vs. post-training and stroke pre- vs. post-training) for each walking condition and 2) within each walking condition across groups (e.g., stroke FW vs. healthy FW). We performed a non-parametric significance test (Kruskal–Wallis) using RStudio software (RStudio Version 2023.12.1, Boston, MA, USA) to test for significant differences where significance was defined as a *p*-value < 0.05. It was determined that the number of synergies for backward walking (BW) compared to backward tandem walking (BT-W) for both the healthy and stroke groups were found to be significant (*p* = 0.01 and 0.008, respectively). All other comparisons were not found to be statistically significant.

## 4. Discussion

Here, we examined if changes in muscle synergies (e.g., number of synergies and muscle recruitment) could facilitate elucidation of how balance training interventions affect those at high fall risk (i.e., older adults that consider themselves overall healthy and those that have survived stroke). In order to direct goal-focused movements, such as overground walking, the central nervous system must map sensory inputs to motor output. Muscle synergies allow insights into how the muscles work together to execute a specific task. Here, the coordinated recruitment groups of specific leg muscles with specific activation balances and specific activation waveforms were observed. The flexible combination of a number of muscle synergies led to a simplified selection of the appropriate muscle commands for a given behavioral goal (overground walking in older adults).

Our study aimed to determine if muscle synergies could be used to quantify changes in high fall-risk groups (healthy older adults and older adults that had suffered a stroke) post-balance training. Within each group (healthy and stroke), muscle activations and muscle synergies were quantified, and some significant changes were observed. Previous studies have suggested that muscle synergy analysis indicates that changes in muscle activation patterns are due to the recruitment of fewer muscle synergies compared to healthy individuals [1]; we observed this here in that we saw increases in muscle activation post-training with corresponding decreases (on average) for the number of muscle synergies. The number of muscle synergies lends insight into the complexity of neuromuscular control needed/required while performing a specific task (i.e., a lower number of synergies equates to a more simplified control strategy). Therefore, decreases in muscle synergies used to execute walking could indicate a better ability for the participants to perform a certain task.

There are some limitations that should be acknowledged in our study. Firstly, there was an imbalance in sample size between the healthy versus stroke participant groups, as well as in terms of female versus male participants. This may affect the overall generalizability of our findings. Further, we did not specify a requirement in terms of the type of stroke experienced by participants, just that participants that had suffered a stroke were at least 6 months post-stroke (i.e., in the chronic phase).

In previous research focused on ‘regular’ forward walking, a range of four to eight synergies that accounted for >80% of the variance had been reported by eleven studies [1]. It had been observed that for undisturbed walking, muscle synergies ranged from 5–8 muscle synergies for roughly 91% VAF [14]. When examining the whole gait cycle, seven synergies were observed for >80% VAF (the major muscles involved were the external obliques (EOBs)) [22] and five synergies were observed for >90% VAF; (the major muscles involved were the medial gastrocnemius medialis (MG) and the biceps femoris (BF)) [23]. In our study, we too observed for the healthy group that the right BF and the right MG showed significant increases in activation for FW and BW, and changes were observed post-compared to pre-training. For the stroke group, the right TA showed significant increases post-training for BT-W. Also, for the stroke group, the right BF (FT-W) and left MG (BW) also showed significant increases. Further, for the healthy group the right RF (quad muscle) showed significant increases post-compared to pre-training. It is important to note that in our study we used a tighter threshold than the aforementioned studies, with VAF set at 98%. This combined with measurements of only four muscles per leg (eight total) may have led to our differences in terms of number of muscle synergies observed. Further, the populations we examined here differed from previous studies in that we researched older adults including those that had suffered a stroke. A previous study was arranged for 6 months and found that the muscle synergy did not change but the muscle definition stability improved [24]. Our study was for an approximately 6-week training cycle per participant. We were able to observe changes in the numbers of synergies, on average, pre-versus post-training for some of the walking conditions, and we did observe differences in synergies across the two groups. More specifically, we found a significant difference between the number of synergies post-BW and post-BW-T for both the healthy and the stroke groups, which highlights the challenges of completing these tasks as the number of synergies increase post- to pre-training.

Muscle analysis, in particular synergies, may thus offer the clinician a viewpoint of neural structure underlying motor behaviors [25]. The possibility of diagnosis (i.e., use of synergies to allow quantification of changes in motor deficits and while undergoing rehabilitation) shows promise. Moreover, such information could inform diagnostic tools and evidence-based interventions for the aging population.

## Figures and Tables

**Table 1.** Demographics of healthy older individuals.

Subject	Age	Male/Female	Ethnicity	Fall and/or Fall-Related Injury (Within Past 5 Years)	Dizziness or Vertigo	Ailments	Activities	Vision
H1	78	Female	Caucasian	Yes	No	Poor dorsiflexion in the left foot	Water aerobics and walking	Glasses
H2	65	Female	Caucasian	No	No	No	No	Glasses
H3	70	Female	Caucasian	No	No	Did not provide	Did not provide	No
H4	70	Female	Caucasian	No	No	Unsure	Visits to wellness center and Jazzercise	Glasses
H5	67	Female	Caucasian	No	No	No	Weightlifting, yoga, and hiking	Glasses (for reading only)
H6	67	Female	Caucasian	No	No	No	Walking and yoga	Glasses
H7	71	Female	Caucasian	Yes	Yes	Hearing loss	Weight-bearing exercises and walking	No
H8	70	Male	Caucasian	Did not provide	yes	Chronic disk impairment	1×/week with med ex-trainer	Glasses (for reading only)
H9	66	Female	Caucasian	No	No	Uneven leg strength (self-diagnosed)	Working out with a trainer, walking, and yoga	Glasses (for reading only)
H10	72	Female	Caucasian	No	No	No	Yes	Glasses
H11	63	Female	Caucasian	No	No	No	Walking, gardening, and yoga	No
H12	68	Female	African American	No	No	Arthritis	No	Glasses
H13	63	Female	African American	No	No	No	Yes	Glasses (for reading only)
H14	74	Female	Caucasian	No	No	No	Water aerobics	Glasses
H15	80	Female	Caucasian	No	No	No	Water aerobics	Glasses
H16	63	Female	Caucasian	No	No	No	Water aerobics	No
H17	78	Female	Caucasian	No	No	No	No	Glasses
H18	71	Female	European	No	Periodic	No	Walking, jogging, and biking	No
H19	71	Female	Caucasian	No	No	No	Low-impact aerobics and yoga	Glasses
H20	64	Female	African American	No	No	No	Balance and strength	Glasses
H21	62	Male	African American	No	No	No	1–2×/week of exercise	Blind in one eye
H22	83	Female	Caucasian	Yes	No	Arthritis	Water aerobics	No
H23	68	Male	Caucasian	No	No	No	Stretching, stationary bike, and yoga	Glasses

**Table 2.** Demographics of older individuals that had suffered stroke.

Subject	Age	Male/Female	Type of Stroke/Notes	Time Elapsed Since Stroke (Years)	Ethnicity	Fall and/or Related Injury (Past 5 Years)	Dizziness or Vertigo	Activities
S1	69	Male	Aneurysm of the left internal carotid artery; subarachnoid hemorrhage bleeding in right hemisphere; ankle foot orthosis and a cane occasionally	33	Caucasian	No	No	Walk in mall about 35′, PT work 1 or 2×/month
S2	65	Male	Cerebellar stroke; uses cane; however, is able to walk without it; regularly exercises	3	Caucasian	No	Yes	Daily walking
S3	61	Male	Weakness on left side due to stroke; very active with regular exercise	3	Caucasian	Yes	No	Personal trainer, pilates
S4	56	Male	Left thalamic intraparenchymal hemorrhage; multiple lacunar infarcts, microhemorrhages, small vessel disease	1,3	African American	No	Yes	Yes
S5	81	Female	Suffered a small acute stroke in the high right frontal lobe with no hemorrhage; may have suffered a second stroke but undiagnosed	0.75	Caucasian	Yes	Periodic	Water aerobics

**Table 3.** Healthy participants’ pre-compared to post-training muscle activation for walking conditions (FW, BW, FT-W, BT-W).

Walking Condition												
		FW			BW			FT-W			BT-W	
Muscle	Pre	Post	*p*-Value	Pre	Post	*p*-Value	Pre	Post	*p*-Value	Pre	Post	*p*-Value
RRF	0.74 (0.04)	0.76 (0.06)	0.00007	0.71 (0.13)	0.73 (0.15)	0.04	0.78 (0.11)	0.77 (0.01)	0.89	0.73 (0.02)	0.3 (1.08)	0.31
LRF	0.7 (0.05)	0.76 (0.06)	0.83	0.72 (0.01)	0.78 (0.08)	0.79	0.67 (0.08)	1.05 (0.79)	0.43	0.68 (0.05)	0.71 (0.08)	0.15
RTA	0.75 (0.06)	0.77 (0.04)	0.18	0.73 (0.02)	0.74 (0.06)	0.3	0.76 (0.07)	0.73 (0.03)	0.61	0.74 (0.01)	0.4 (0.73)	0.5
LTA	0.75 (0.05)	0.75 (0.07)	0.1	0.71 (0.06)	0.84 (0.33)	0.48	0.67 (0.06)	0.67 (0.06)	0.62	0.71 (0.03)	1.02 (0.59)	0.08
LBF	0.71 (0.06)	0.73 (0.05)	0.21	0.72 (0.05)	0.72 (0.06)	0.43	0.72 (0.1)	0.68 (0.13)	0.87	0.65 (0.09)	0.32 (0.99)	0.1
RBF	0.64 (0.05)	0.73 (0.05)	0.03	0.7 (0.01)	0.63 (0.11)	0.36	0.72 (0.08)	0.63 (0.10)	0.35	0.68 (0.11)	0.24 (1.07)	0.36
LMG	0.69 (0.07)	0.69 (0.14)	0.06	0.75 (0.08)	0.83 (0.16)	0.36	0.67 (0.13)	0.73 (0.07)	0.87	0.67 (0.06)	1.1 (0.85)	0.18
RMG	0.64 (0.05)	0.75 (0.14)	0.005	0.67 (0.02)	0.8 (0.28)	0.58	0.66 (0.05)	0.58 (0.13)	0.2	0.64 (0.14)	0.67 (0.10)	0.77

Means are shown with standard deviation in parentheses. The color-coding highlights significance level: *p* < 0.05 (blue), *p* < 0.005 (green), *p* < 0.001 (yellow).

**Table 4.** Stroke participants’ pre-compared to post-training muscle activation for walking conditions (FW, BW, FT-W, BT-W).

Walking Condition												
		FW			BW			FT-W			BT-W	
Muscle	Pre	Post	*p*-Value	Pre	Post	*p*-Value	Pre	Post	*p*-Value	Pre	Post	*p*-Value
RRF	0.78 (0.05)	0.79 (0.05)	0.18	0.79 (0.04)	0.76 (0.04)	0.04	0.8 (0.02)	0.8 (0.03)	0.03	0.79 (0.05)	0.7 (0.04)	0.14
LRF	0.79 (0.03)	0.79 (0.02)	0.29	0.81 (0.05)	0.7 (0.07)	0.40	0.76 (0.02)	0.73 (0.1)	0.19	0.76 (0.02)	0.66 (0.03)	0.26
RTA	0.7 (0.03)	0.75 (0.04)	0.15	0.69 (0.05)	0.81 (0.03)	0.06	0.65 (0.03)	0.74 (0.07)	0.14	0.63 (0.02)	0.72 (0.05)	0.02
LTA	0.71 (0.06)	0.74 (0.03)	1	0.69 (0.06)	0.61 (0.05)	0.09	0.63 (0.04)	0.78 (0.09)	0.19	0.59 (0.07)	0.67 (0.06)	0.20
LBF	0.73 (0.06)	0.81 (0.04)	0.34	0.76 (0.04)	0.85 (0.04)	0.08	0.71 (0.04)	0.8 (0.04)	0.07	0.7 (0.05)	0.83 (0.03)	0.14
RBF	0.68 (0.06)	0.76 (0.04)	0.56	0.66 (0.06)	0.74 (0.06)	0.11	0.61 (0.05)	0.7 (0.05)	0.3	0.63 (0.07)	0.71 (0.08)	0.33
LMG	0.71 (0.05)	0.77 (0.03)	0.22	0.73 (0.04)	0.76 (0.05)	0.02	0.68 (0.04)	0.75 (0.07)	0.07	0.67 (0.03)	0.79 (0.06)	0.07
RMG	0.68 (0.06)	0.75 (0.05)	0.22	0.63 (0.06)	0.75 (0.11)	0.12	0.61 (0.05)	0.65 (0.06)	0.60	0.56 (0.07)	0.65 (0.11)	0.87

Means are shown with standard deviation in parentheses. The color-coding highlights significance level: *p* <0.05 (blue).

**Table 5.** Cohen’s d results for pre- vs. post-training for stroke participants in all walking conditions. Small (red), medium (blue), and large (green) effect sizes are noted in the table; a negative symbol is shown to indicate directionality.

Cohen’s *d*				
Muscle	Walking Forward	Walking Backward	Tandem Forward	Tandem Backward
	Pre vs. Post	Pre vs. Post	Pre vs. Post	Pre vs. Post
RRF	0.2	0.75	0.0	−1.9
LRF	0.4	1.9	−0.5	−3.2
RTA	1.1	2.6	1.7	2.1
LTA	0.0	−1.4	2.5	1.2
LBF	1.1	2.3	2.0	2.8
RBF	1.5	1.3	1.5	1.1
LMG	0.8	0.7	1.3	2.6
RMG	1.0	1.4	0.6	0.9

**Table 6.** Healthy group’s number of muscle synergies for 80%, 90%, and 98% VAF with constant confidence interval of 90%.

VAF	Walking Condition							
	FW		BW		FT-W		BT-W	
	Pre	Post	Pre	Post	Pre	Post	Pre	Post
80%	1.00	1.00	1.05	1.00	1.03	1.00	1.00	1.00
90%	1.31	1.27	1.23	1.11	1.30	1.17	1.19	1.19
98%	3.02	3.00	3.25	3.06	3.41	3.26	3.65	3.42

**Table 7.** Stroke group’s number of muscle synergies for 80%, 90%, and 98% VAF with constant confidence interval of 90%.

VAF	Walking Condition							
	FW		BW		FT-W		BT-W	
	Pre	Post	Pre	Post	Pre	Post	Pre	Post
80%	1.00	1.00	1.14	1.00	1.00	1.00	1.00	1.00
90%	1.25	1.14	1.14	1.00	1.50	1.33	1.33	1.00
98%	2.38	2.71	3.29	3.00	4.00	3.00	3.67	4.00

**Table 8.** Healthy and stroke groups’ muscle synergies (98% VAF), pre-compared to post-training for walking conditions (FW, BW, FT-W, BT-W). Means are shown with standard deviation in parentheses.

Group	Walking Condition							
	FW		BW		FT-W		BT-W	
	Pre	Post	Pre	Post	Pre	Post	Pre	Post
Healthy	3.02(0.84)	3.00(0.81)	3.25(0.75)	3.06(0.82)	3.41(0.82)	3.26(0.63)	3.65(0.58)	3.42(0.73)
Stroke	2.38(0.74)	2.71(0.95)	3.29(1.50)	3.00(0.00)	4.00(1.51)	3.00(0.00)	3.67(1.03)	4.00(0.00)

## Data Availability

The raw data supporting the conclusions of this article will be made available by the authors on request.

## Abbreviations

The following abbreviations are used in this manuscript:

sEMG: surface electromyography
FW: forward walking
FT-W: forward tandem walking
BW: backward walkin
BT-W: backward tandem walking
RF: rectus femoris
TA: tibialis anterior
BF: bicep femoris
MG: medial gastrocneumius
